# Cerebral networks of apathy and goal-oriented patterns of actimetry in late-life depression

**DOI:** 10.1192/j.eurpsy.2023.53

**Published:** 2023-07-19

**Authors:** G. Robert

**Affiliations:** ^1^Academic old-age psychiatry, Centre Hospitalier Guillaume Régnier; ^2^Empenn, Institut de Recherche en Informatique et Systèmes aléatoires (IRISA), Rennes, France

## Abstract

**Abstract:**

Apathy and goal-oriented behaviors are key dimensions of late-life depression (LLD) and are iteratively associated with cognitive decline in most neuropsychiatric disorders. However, scales and criteria remains insufficient to provide robust individual biomarkers that could foster personalized therapeutic approaches. Therefore, dimensional and digital phenotyping offer new possibilities of stratifying LLD population. This presentation will show our recent results of functional connectivity cerebral networks associated with multidimensional paper-and-pen measures of apathy, including the default mode and the cingulo-opercular networks. We will also show how dimensional reduction (functional principal component analysis) of 3 days actimetry measures are associated with both FC and inflammatory measures (diffusion and multicompartment indices such as free water and neurite orientation dispersion), providing arguments for different pathophysiological mechanisms underlying goal-oriented behaviors and reinforcing actimetry as a good candidate for individual biomarker of cognitive decline in LLD. Finally, machine learning approaches using combination of different, yet correlated, indices of actimetry will be presented and discussed with their corresponding classifying accuracies (outside of cerebral imaging). Altogether, this presentation aims at bridging the gap between cerebral imaging and digital phenotyping to enhance personalized medicine in the field of old-age psychiatry and cognitive decline prevention.

**Disclosure of Interest:**

None Declared

